# Adherence to inhaled corticosteroids by asthmatic patients: measurement and modelling

**DOI:** 10.1007/s11096-013-9862-0

**Published:** 2013-12-01

**Authors:** Amelia Taylor, Li-Chia Chen, Murray D. Smith

**Affiliations:** Division for Social Research in Medicines and Health, School of Pharmacy, The University of Nottingham, University Park, Nottingham, NG7 2RD UK

**Keywords:** Adherence, Asthma, Exacerbation, Panel data, Prescription possession ratio

## Abstract

*Background* Poor adherence to inhaled corticosteroids (ICS) is known as the main cause for therapeutic failure in asthma treatment and associated morbidity. To improve adherence, targetted and effective interventions need to be developed ideally based on using longitudinal follow-up of a large study cohort to establish patterns and influences on adherence. *Objective* To develop an annual measure of asthma patients’ adherence to ICS using primary care prescribing data over consecutive annual intervals, and to statistically model ICS adherence controlling for a range of patient factors. *Setting* A retrospective cohort study between 1997 and 2010 using United Kingdom general practice prescribing data on asthma patients aged between 12 and 65 years, without a diagnosis of chronic obstructive pulmonary disease. *Method* Patient’s ICS prescriptions are used to calculate the ‘number of days prescribed during calendar year’ divided by ‘number of days in the interval’ to form an annual prescription possession ratio (PPR) for each patient. Several definitions of PPR are considered and compared when calculating numerator and denominator. Adherence, measured by the preferred PPR, is then modelled to estimate the effect of asthma exacerbation, severity, control and other patient factors on adherence. *Main outcome measure* PPR, being a proxy measure for adherence. *Results* Annual PPR by all strategies gave a similar frequency profile. ICS were either over- or under-prescribed for over half of the follow-up time. Adherence was lower in younger patients, those newer to the study timeframe, those with less severe asthma, those with good control, with lower previous adherence, and who had not previously experienced an exacerbation. *Conclusion* The chosen PPR simulated clinical use of ICS most closely; including overlapping days, excess days passed to the next interval, considering gaps in the denominator, with censoring at 100 %. The PPR is a useful measure for signalling or measuring adherence changes over time. The modelling results identified many characteristics which would indicate which asthma patients and at what points in their treatment cycle they would be at increased risk of low adherence.

## Impact of findings on practice statements

- A method to measure adherence has been developed for use with ICS in asthma patients, which could be used in other retrospective adherence studies or in clinical practice to monitor patients’ adherence.
- Modelling methodology has begun to identify risk factors that could be used to target asthma patients at risk of poor adherence to ICS, which could be used to identify and target efficient and effective interventions to improve adherence.

## Introduction

Medication adherence is commonly defined as: “the extent to which a patient acts in accordance with the prescribed interval and dose of a dosing regimen” [1]. Non-adherence to medicine is associated with reduced health outcomes [2–4] and is a notable issue for long-term conditions as non-adherence rates for long-term therapy typically exceed 50 % [5–7]. A better understanding of the patterns of change in adherence over time and the factors that may contribute to poor adherence is especially important in the management of long term chronic conditions. In order to elicit these patterns and influences, longitudinal follow-up and large sample sizes are needed to facilitate the necessary statistical analyses. Outcomes from such work will contribute to the development of efficient adherence-promoting interventions targeted at the most appropriate times in a patient’s treatment cycle.

Currently, there is no gold standard measure of adherence. Approaches to measurement include pill counts, electronic measuring devices, patient log books etc. These may be suitable in small clinical trials but are impractical and overly expensive for monitoring medicine use during routine care, or across large samples of patients. Dispensing data administratively linked to medical records have been used in some studies [8] but in the UK such data are not available in sufficiently large samples. Alternately, patient-level primary care prescribing data can be used to generate a prescription possession ratio (PPR), defined as the proportion of the combined number of days prescribed by individual prescriptions over an annual interval as part of a patient’s long term treatment. PPR can be considered a proxy measure for adherence under the assumption that a patient will fill prescriptions and take the medicine. However, prescribing frequency and quantity are affected by both patient attendance at a doctor’s appointment to be able to receive the prescription (or to request a repeat prescription) and the prescribers choice to write the prescription. Despite these limitations, evidence from a New Zealand study (n = 646) found that adherence estimated using prescription data was “a useful predictor of dispensing-based adherence” [9].

For asthma management, although the evidence-based effectiveness of asthma medicines has been proven and clear prescribing guidelines are available [10, 11], adherence to asthma medicines is known to be low; for example, Andersson et al. [12] found refill-adherence for asthma medicines one of the lowest (34 %) when compared to treatments for other long-term conditions.

In this study, we focussed on inhaled corticosteroids (ICS) because adherence by asthmatics to these medicines has been reported to be especially poor [13] and has been identified as the main cause for failure in asthma treatment [14] with consequences that include increases in asthma exacerbations, decreases in patient quality of life [6], and increases in morbidity, mortality and healthcare costs to the UK’s National Health Service [15, 16]. The effect of different factors on adherence were considered, including the age of the patient, control of symptoms, severity of asthma, and whether the patient had experienced an asthma exacerbation.

## Aim of the study

First, to develop an annual measure of asthma patients’ adherence to ICS by using UK primary care prescribing data over consecutive annual intervals; and second, to construct and estimate a patient-level statistical model of ICS adherence controlling for a range of individual patient factors.

## Methods

### Study design and cohort

This retrospective cohort study used data extracted from the Clinical Practice Research Datalink (CPRD) database [17]. CPRD is a longitudinal database containing anonymised medical records on approximately 12.5 million acceptable patients (December 2012 build) registered across 661 general practices located throughout the UK (April 2013 build). Included in the study were asthma patients aged between 12 and 65 years whose records in CPRD fell within the study period 1997–2010 and who were consented for administrative linkage to their hospital episode statistics (HES) secondary care inpatient records, and who were without chronic obstructive pulmonary disease (COPD). Patients were followed from their respective index date (when the patient met the inclusion criteria for entry into the sample frame) up until either: (a) the end date of the study, or (b) when they reached their 65th birthday, or (c) were diagnosed with COPD, or (d) died or were transferred out of their GP practice. Approval for the study was granted by the Independent Scientific Advisory Committee of the Medicines and Healthcare products Regulatory Agency (protocol number 13_036R).

### Data management

Included patients’ ICS prescriptions were collected and the prescribing date and duration (number of days prescribed) were then used to calculate PPR. Missing values for prescribing duration were imputed by calculating the number of doses prescribed (quantity of packs multiplied by its number of doses) divided by the recorded daily prescribed dose. Errors (such as duplications, swaps, or missing values) in the number of doses in the pack (pack type) were checked and any outlying values were corrected based on pack information taken from the British National Formulary [18]. Missing values for the daily prescribed dose were imputed using the patient’s prior prescription records or, if unavailable, by substitution of the sample median of the daily prescribed dose by dosage form.

### Asthma exacerbation and severity of asthma

Asthma exacerbations were distinguished by whether they were hospital-recorded (the primary diagnosis ICD-10 coding in HES episode data was J45 or lower) or were managed in primary care (identification of oral prednisolone use to treat exacerbation in the CPRD therapy file). In addition, keywords (“asthma” and “exacerbation”, “emergency prednisolone”, “admit to hospital”, etc) were matched with relevant Read codes to identify occurrences of them in the CPRD clinical file, which were then classified as exacerbation treatment within primary or secondary care.

To identify oral prednisolone prescribing to treat an exacerbation, criteria considering the duration (less than 10 days per prescription, less than 90 days per year) and quantity/dose (qty of less than or equal to 20 and strength is 25 mg, or qty of less than or equal to 112 and strength is 5 mg) were used. Any patient-years with a prednisolone prescription which failed to meet these criteria were not considered to be indicative of an exacerbation. Instead, these patient-years were classified as being treated within step 5 of the British Thoracic Society and Scottish Intercollegiate Guidelines Network (BTS/SIGN) guidelines [11]. These guidelines are such that a patient’s asthma severity is increasing in treatment step (ranging from steps 1 to 5) where additional medicines or higher doses are required to achieve control. A patient presenting at step 1 has their asthma controlled with a short-acting β_2_-agonist (SABA) alone, whereas a step 5 patient requires routine daily prednisolone treatment in order to control their asthma.

### Measuring adherence

In this study, a patient’s adherence to ICS prescriptions was measured using PPR. This was calculated by dividing ‘number of days prescribed during calendar year’ by ‘number of days in the interval’ and thus was the proportion of days in the year where medicine was prescribed [8]. Expressed as a percentage, it was constructed as follows:

$$ PPR = 100 \times \frac{{{\text{Number}}\,\,{\text{of}}\,\,{\text{days}}\,\,{\text{prescribed}}\,{\text{during}}\,\,{\text{calendar}}\,\,{\text{year}}}}{{{\text{Number}}\,\,{\text{of}}\,\,{\text{days}}\,\,{\text{in}}\,\,{\text{the}}\,\,{\text{interval}}}} $$

**Fig. 1:**
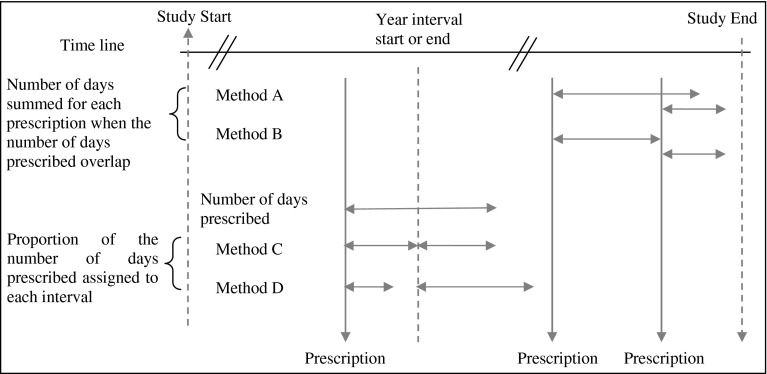
Calculating the PPR numerator when prescription supply overlaps or when the prescription cuts across the interval start or end

Several approaches were considered when evaluating the numerator, distinguished by whether or not to include or exclude the overlap in prescription days, and whether to pass excess prescription days over to the next interval or to share these proportionally between intervals (see Fig. 1). The denominator was set to 365 days for an annual interval, but was adjusted at the beginning or end for when a patient entered or left the follow-up, or for missing data in number of doses.

By combining these approaches in differing ways, four possible strategies were defined for calculating PPR (see top half of Table 1), of which the first—strategy 1 (including overlapping days, passing excess days to the next interval, and adjustments to the beginning and end intervals)—represents the base case. A fifth strategy imposed a censoring rule on the base case, namely, any computed value of PPR that exceeded 100 % is reset to 100 %. PPR was calculated for each patient annually using each strategy (1–5) and the results presented descriptively.

**Table 1:** Descriptive statistics

PPR strategy^a^		Patient-years (total = 824,943)	Mean	SD	Min	Max	Median
1	A, C, E	822,503	85.45	71.51	0.46	6,135.71	69.86
2	B, C, E	822,503	61.52	27.02	0.36	100	61.92
3	A, D, E	662,797	87.86	67.37	0.36	4,718.52	73.47
4	C, C, F	822,526	80.23	58.28	0.82	1,362.74	66.3
5	Censored strategy 1 at 100 %	822,503	57.56	28.92	0.46	100	69.86
*Patient characteristics*							
	Age in years		38.72	15.41	12	65	39
	Years in study		3.90	2.96	1	14	3
	SABA use		0.04	0.19	0	1	0
	BTS/SIGN step		2.70	0.85	2	5	2
	Annual change in step		0.02	0.45	−3	3	0
*Asthma exacerbations*							
	Annual total		0.29	0.84	0	19	0
	Hospital admission		0.01	0.08	0	1	0
	Primary care management		0.18	0.38	0	1	0

^a^A: including overlapping days; B: excluding overlapping day; C: pass excess days to next interval; D: share excess days proportionally between intervals; E: interval set as 365 days; F: adjusted the beginning and end intervals

### Modelling adherence

Prescription possession ratio serves as a proxy variable for adherence (we denote adherence by *A* and note that it is unobservable) and so it is subject to error when using it as a measure of adherence. Let the relationship between these variables be:

$$ PPR = A + U $$

where the unobservable error term *U* is assumed to have zero mean. We constructed and estimated inferential models designed to explain adherence *A* in terms of its dynamic behaviour through time as well as to demonstrate its relationship to clinical outcomes and other factors. In particular, we use a panel data model to match the study’s data structure (unbalanced panel data) and consider fixed effects representations, for example:

$$ PPR_{it} \,= \,\alpha PPR_{i,t - 1} + \gamma Y_{it} + \beta_{0} + \beta_{1} X_{1it} + \cdots + \beta_{k} X_{kit} + \lambda_{i} + U_{it} $$

where indexes $i = 1, \ldots ,N$ patients and $t = 1, \ldots ,T$ time periods. The unknown coefficients to be estimated include: $\alpha$ the coefficient of the lagged dependent variable $PPR_{ - 1}$ designed to capture any dynamic relationships adherence may have with itself over time; $\gamma$ the coefficient of *Y* that represents clinical outcome and which arguably has a feedback causal effect with adherence; $(\beta_{0} ,\beta_{1} , \ldots ,\beta_{k} )$ as the set of $k + 1$ coefficients on a set of independent regressors $(1,X_{1} ,X_{2} , \ldots ,X_{k} )$ where 1 denotes the intercept and $(X_{1} ,X_{2} , \ldots ,X_{k} )$ are patient-measured attributes (see Table 2). The patient-specific, arbitrarily distributed individual fixed effect is represented by $\lambda$. The error term *U* can be heteroscedastic and autocorrelated. This type of panel data model has been extensively studied and applied in numerous studies [19–21]. System generalised method of moments (system GMM) is an appropriate estimator for fixed effects models with a mix of lagged dependent variables, endogenous and predetermined regressors as well as strictly exogenous regressors. We implemented this estimator using Roodman’s xtabond2 algorithm [22] which is an add-on to the STATA software.

**Table 2:** Data definitions

Patient characteristics	Description
Age in years	The age of the patient in years
Years in study	The number of years since the patient first met the inclusion criteria for the study
SABA use	Indicator of asthma control. Prescribing of over 10 doses per day on average over the year indicates poor control (1 = patient has received prescriptions for over 10 SABA per day)
BTS/SIGN step	Indicator of asthma severity. The treatment step taken from the British Guideline on the Management of Asthma [11]. Patients treated within steps 2–5 are included in the study
Annual change in step	Indicator for whether a patient has increased or decreased in severity from their previous year in the study
Annual total asthma exacerbations	Number of asthma exacerbations in the year
Hospital admission	Asthma exacerbation requiring a hospital admission (1 = the patient has been admitted at least once in the year for asthma exacerbation)
Primary care management	Asthma exacerbation treated within primary care (1 = the patient has been treated within primary care at least once in the year for asthma exacerbation)

## Results

### Measuring adherence

Overall, 292,738 patients with 1,181,033 patient-years of data were included in this study. Descriptive statistics for each PPR measurement strategy are listed in the top half of Table 1. By design, strategies 2 and 5 had a maximum possible PPR value of 100 %, while for those strategies in which PPR can exceed 100 %, similar extreme ranges were observed. For the base-case (strategy 1), there were 28.2 % of patient-years with PPR that exceeded 100 %, while 32.0 % of patient-years had PPR values lower than 50 %. The proportions of patient-years below 20 % were consistent across all five strategies.

**Fig. 2:**
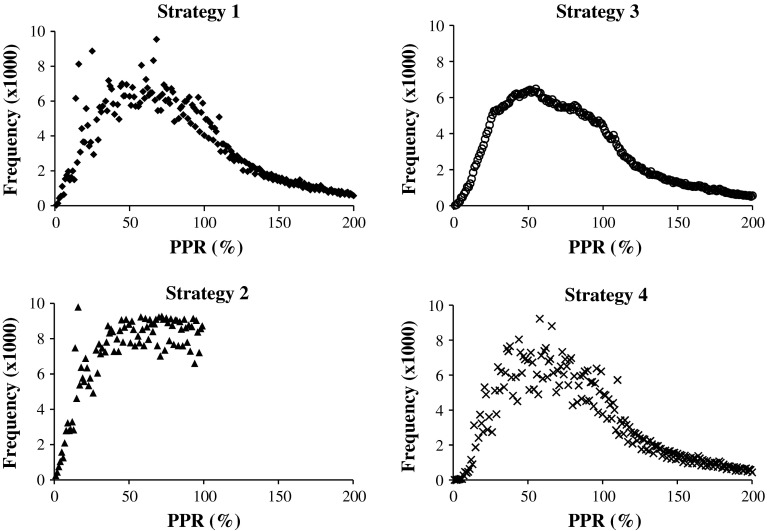
PPR empirical relative frequency function

The empirical relative frequency functions of all PPR measurements are depicted in Fig. 2 for each of strategies 1–4.

Since all of the methods had a similar frequency profile for PPR, the strategy considered to be the most clinically appropriate was selected to take forward for modelling purposes. In clinical practice, patients are unlikely to discard remaining doses when they receive a new prescription or at the end of a year, especially if the new prescription is for the same medicine. It is likely that patients will have gaps in prescribing due to missing data or when newly entering into, or leaving their registration at their general practice. Further, once a patient receives enough medicine to cover every day of the year, they should receive no additional clinical benefit from any additional doses prescribed. These practices are most closely simulated by strategy 5, therefore it was this measure which we used to proxy adherence to ICS and which was subsequently used for modelling purposes.

### Modelling adherence

Using PPR measured using strategy 5, three sets of estimation results are presented in Table 3: for all patients (column 1) and gender-specific in columns (2) and (3). The periodicity in the model was annual and as each fitted model contains 2 prior lags of *PPR*, only patients recording data in 3 or more years were included in the estimation runs; patient numbers are reported under *N*. Estimates and associated t-statistics (ratio of estimate to standard error) are reported. Regression controls were allocated into three major categories: patient characteristics, persistence and asthma exacerbations. Baseline values for categorical variables are indicated by zero coefficients, and so results can be interpreted relative to a patient who is at BTS/SIGN treatment step 2 with no high use of SABA in the current year and who has had no exacerbations of either type in the prior year. Note that descriptive statistics associated with the controls were reported earlier in the bottom half of Table 1.

**Table 3:** System GMM estimation results

	(1)		(2)		(3)	
	All		Male		Female	
Patient count *N*	97,456		42,740		54,716	

	Estimate	T-stat	Estimate	T-stat	Estimate	T-stat
Intercept	24.757	65.02	25.725	41.62	23.892	49.85
*Patient characteristics*						
Age in years	0.110	37.38	0.108	25.24	0.115	28.03
Years in study	0.257	20.56	0.249	12.80	0.264	16.02
SABA use, no = 0	0		0		0	
SABA use, yes = 1	5.108	29.55	4.898	20.58	5.380	21.74
BTS/SIGN step 2	0		0		0	
BTS/SIGN step 3	−1.472	−13.79	−1.578	−9.96	−1.353	−9.42
BTS/SIGN step 4	−2.513	−20.19	−2.551	−14.66	−2.365	−13.91
BTS/SIGN step 5	−4.014	−8.62	−2.126	−2.81	−4.868	−8.28
Annual change in step	2.595	31.37	2.247	17.60	2.799	25.83
*Persistence*						
*PPR*_−1_	0.503	141.89	0.500	88.13	0.507	111.05
*PPR*_−2_	0.102	34.82	0.099	21.52	0.103	27.06
*Exacerbations*						
Total in current year	0.241	1.43	0.655	2.71	−0.090	−0.42
Prior year hospital admission, no = 0	0		0		0	
Prior year hospital admission, yes = 1	1.653	2.04	2.511	2.11	0.735	0.67
Prior year primary care, no = 1	0		0		0	
Prior year primary care, yes = 1	0.942	6.90	1.064	4.71	0.912	5.31
Prior year interaction	−0.378	−0.34	−1.024	−0.58	0.989	0.68

Baseline values for categorical variables are indicated with a zero coefficient: being a patient at BTS/SIGN treatment step 2 with no high use of SABA in the current year and no exacerbations of either type in the prior year

#### Persistence

The positive, statistically significant estimates of the coefficients of lagged *PPR* (*PPR*_−1_ is current *PPR* lagged by 1 year, and *PPR*_−2_ lags it by 2 years) show that the patient’s history of adherence behaviour has a strong and reinforcing influence on their current attitude towards ICS adherence.

#### Characteristics

Adherence increases with patient age (+0.11 %/year; t = 37.4) as too it trends positively the longer the patient remains in contact with the prescriber (years in study: +0.26 %/year; t = 20.6). High SABA use is indicative of poor asthma control over a sustained period, when SABA was used in this manner our estimation results indicated that adherence to ICS was significantly boosted by approximately 5 % alongside it (+5.1 %; t = 29.6). As might be expected adherence worsened as patients were treated at higher steps in the BTS/SIGN guidelines (step 2 is the base); however, it is important to note the modification that should an annual worsening in asthma status occur (i.e. ‘annual change in step’ = 1 or more), it prompted the patient to reconsider their adherence behaviour and improve it +2.6 % per increment (t = 31.4). The flip side to this was that a patient presenting with an annual improvement in their asthma status (i.e. ‘annual change in step’ = −1 or lower) was expected to worsen in adherence.

#### Exacerbations

The positive estimate (+0.241 %/attack; t = 1.43) implied that adherence improved as the number of exacerbations (hospital admit plus managed in primary care; assumed endogenous) increased, although the estimate did not significantly differ from zero. Included into the model and separated by destination of care were indicators of exacerbation occurrence in the previous year; these variables were predetermined. The evidence was now statistically stronger: patient’s behavioural response to the occurrence of past exacerbations was to increase their adherence to ICS, where the +1.653 % estimate (t = 2.04) of the adherence effect was greater than +0.942 % (t = 6.9) than if all prior year exacerbations were managed solely in primary care.

#### Gender differences

The differences that emerged when stratifying by gender focussed mainly on response to exacerbations. As the total number of current year exacerbations increased males responded by improving their decision to adhere to ICS whereas amongst females this was insignificant (males: +0.655 %/attack; t = 2.71 vs females: −0.09 %/attack; t = −0.42). Also in evidence is the response to prior year hospital-treated exacerbation, where the response by males sees a significant improvement in their current decision to adhere to ICS (+2.511 %; t = 2.11), whereas amongst females their response is insignificant (+0.735 %; t = 0.67).

## Discussion

Prescription possession ratio results were not greatly affected by the method chosen, except for the censoring or restricting of measures to a maximum of 100 % in strategies 2 and 5. Larger variances were reported for strategies 1, 3 and 4, caused by outliers but mainly by large numbers of values generated in excess of 100 % (observed to be 28.4 % of patient-years). By censoring these at 100 %, the mean and variance of PPR must decrease. The presence of under-prescribing was also highlighted (observed to be 32.1 % of patient-years in which PPR < 50 %). The clinical reasons for both eventualities, over- and under-prescribing, warrants further investigation. Amongst possible reasons for over-prescribing may be an absence of understanding by the prescriber about what has been prescribed previously, or by patients receiving but not filling prescriptions. Under-prescribing may be caused by the patient’s desire to avoid taking the medicine, by the physician or the patient deeming it to be appropriate despite not being in line with treatment guidelines [4].

The modelling results reinforce prior expectations that the better was a patient’s health the lesser was the incentive for them to adhere to a long-term ICS regimen. On the other hand, the model showed that when adverse exacerbation events occurred, patients’ ICS adherence would, at least on average, be forcibly improved. Moreover, younger age remained a significant factor detrimental to adherence for both sexes; for example, the ICS adherence prediction from the fitted model for a 16 year-old with step 2 disease averages barely more than 25 %. Admittedly, ‘high’ concomitant use of SABA increased the 16 year-olds’ prediction to approximately 30 %, but even so when coupled with evidence of strong behavioural persistence over time the young asthmatic was expected to be non-adherent to ICS for some years, and therefore should arguably be a prime target for adherence-promoting policy interventions.

### Strengths and limitations

Other studies have found PPR to be reflective of the medicine possession ratio (MPR) [23]; a measure of adherence frequently used in studies using prescription fill data rather than prescribing data. PPR can be a very useful tool for measuring adherence using the very rich source of retrospective data available in the UK, however, it is difficult to interpret the accuracy of the measure without comparing the adherence measured by PPR against adherence measured directly; but, the precision of the method appears to be good. Therefore, PPR should be used with caution to determine actual levels of adherence, but if used can be very valuable to measure changes or differences in adherence over time.

The use of the CPRD prescribing data to calculate adherence, with its large rich source of clinical and patient characteristic data, allows the impact of other patient and clinical characteristics that may affect adherence to be considered. However, there are several known limitations of using retrospective databases for analysis that would be expected to affect some of the patient records; including missing or incorrectly recorded information and incentives giving rise to record specific types of data and which assign lower priority to others. Despite these limitations, the large data source allows the impact on conclusions of these limitations (as long as they are considered in study design) to be minimal.

There are also many factors that could affect adherence, but cannot be measured in this setting. Examples of these would be patient attitudes to their condition or medicine, or the reasons for the decisions taken by the health care professional to prescribe ICS. The PPR measure uses the assumption that patients should be prescribed a regular daily dose of ICS to treat their asthma; however the intention of the health care professional to prescribe a daily dose is not available. If unobserved heterogeneity is integral to the study outcomes, then it would need to be considered in any modelling of the data and in the inferences drawn. While the use of fixed effects in modelling goes some way towards mitigating the deleterious effect of unobserved heterogeneity, it is still only a partial solution.

## Conclusion

An annual measure of asthma patients’ adherence to ICS, the PPR, measured using CPRD data, was constructed and found to be a useful measure for signalling, or measuring adherence changes over time. The chosen PPR methodology simulated clinical use of ICS most closely; including overlapping days, excess days passed to the next interval, considering gaps in the denominator, with censoring at 100 %. The methods for calculating PPR could be applied to other chronic conditions; however the method chosen must be based on knowledge of the specific clinical setting and disease-medicine characteristics.

A patient-level statistical model of ICS adherence was constructed, controlling for a range of individual patient factors. The modelling results identified many characteristics which would indicate which asthma patients and at what points in their treatment cycle they would be at increased risk of low adherence. These risk factors included those with poor adherence in the previous year, younger patients, higher treatment step, and those patients who have not recently experienced an exacerbation.
